# Supplementary material for: Decentralised trials for hearing and tinnitus therapies: Lessons from the Digital thErapy For Improved tiNnitus carE (DEFINE) randomised controlled trial
Source: PLoS One. 2025 Aug 14;20(8):e0324927. doi: 10.1371/journal.pone.0324927 (PMC12352653; doi:10.1371/journal.pone.0324927)
Supplement: Attachment — Submitted filename: Rebuttal Letter for PLOS One.docx [file pone.0324927.s003.docx]

1. Please ensure that your manuscript meets PLOS ONE's style requirements, including those for file naming. The PLOS ONE style templates can be found at

<https://journals.plos.org/plosone/s/file?id=wjVg/PLOSOne_formatting_sample_main_body.pdf> and

[https://journals.plos.org/plosone/s/file?id=ba62/PLOSOne_formatting_sample_title_authors_affiliations.pdf](https://track.editorialmanager.com/CL0/https:%2F%2Fjournals.plos.org%2Fplosone%2Fs%2Ffile%3Fid=ba62%2FPLOSOne_formatting_sample_title_authors_affiliations.pdf/1/010f0195882786d5-85ee1977-8e8c-4401-aec2-f85b47854d50-000000/btQ4GYp18a5mWhQuUlGW1Chvs70Ao0H7u5tlD4O7Kb0=202)

**RESPONSE**: We have checked that our manuscript and it follows the PLOS One Style requirements.

2.  We note that your Data Availability Statement is currently as follows: “All relevant data are within the manuscript and its Supporting Information files.”

Please confirm at this time whether or not your submission contains all raw data required to replicate the results of your study. Authors must share the “minimal data set” for their submission. PLOS defines the minimal data set to consist of the data required to replicate all study findings reported in the article, as well as related metadata and methods (<https://journals.plos.org/plosone/s/data-availability#loc-minimal-data-set-definition>).

For example, authors should submit the following data:

- The values behind the means, standard deviations and other measures reported;
- The values used to build graphs;
- The points extracted from images for analysis.

Authors do not need to submit their entire data set if only a portion of the data was used in the reported study.

If your submission does not contain these data, please either upload them as Supporting Information files or deposit them to a stable, public repository and provide us with the relevant URLs, DOIs, or accession numbers. For a list of recommended repositories, please see <https://journals.plos.org/plosone/s/recommended-repositories>.

If there are ethical or legal restrictions on sharing a de-identified data set, please explain them in detail (e.g., data contain potentially sensitive information, data are owned by a third-party organization, etc.) and who has imposed them (e.g., an ethics committee). Please also provide contact information for a data access committee, ethics committee, or other institutional body to which data requests may be sent. If data are owned by a third party, please indicate how others may request data access.

**RESPONSE**: We can confirm all relevant data is included. This is confirmed by both Reviewers who responded YES to the question “Have the authors made all data underlying the findings in their manuscript fully available?”.

3. Please include your full ethics statement in the ‘Methods’ section of your manuscript file. In your statement, please include the full name of the IRB or ethics committee who approved or waived your study, as well as whether or not you obtained informed written or verbal consent. If consent was waived for your study, please include this information in your statement as well.

**RESPONSE**: This has now been inserted into the Methods section as “). NHS ethical approval was granted by the West Midlands - Black Country Research Ethics Committee (REC Reference: 23/WM/0146) with all participants providing written consent to be involved in the study.”

4. Please include a separate caption for each figure in your manuscript.

**RESPONSE**: These have been added to the manuscript

5. We note that Figure 1 and 2 in your submission contain copyrighted images. All PLOS content is published under the Creative Commons Attribution License (CC BY 4.0), which means that the manuscript, images, and Supporting Information files will be freely available online, and any third party is permitted to access, download, copy, distribute, and use these materials in any way, even commercially, with proper attribution. For more information, see our copyright guidelines: <http://journals.plos.org/plosone/s/licenses-and-copyright>.

We require you to either (1) present written permission from the copyright holder to publish these figures specifically under the CC BY 4.0 license, or (2) remove the figures from your submission:

A. You may seek permission from the original copyright holder of Figure 1 and 2 to publish the content specifically under the CC BY 4.0 license.

We recommend that you contact the original copyright holder with the Content Permission Form (<http://journals.plos.org/plosone/s/file?id=7c09/content-permission-form.pdf>) and the following text:
“I request permission for the open-access journal PLOS ONE to publish XXX under the Creative Commons Attribution License (CCAL) CC BY 4.0 (<http://creativecommons.org/licenses/by/4.0/>). Please be aware that this license allows unrestricted use and distribution, even commercially, by third parties. Please reply and provide explicit written permission to publish XXX under a CC BY license and complete the attached form.”

Please upload the completed Content Permission Form or other proof of granted permissions as an "Other" file with your submission.

In the figure caption of the copyrighted figure, please include the following text: “Reprinted from [ref] under a CC BY license, with permission from [name of publisher], original copyright [original copyright year].”

B. If you are unable to obtain permission from the original copyright holder to publish these figures under the CC BY 4.0 license or if the copyright holder’s requirements are incompatible with the CC BY 4.0 license, please either i) remove the figure or ii) supply a replacement figure that complies with the CC BY 4.0 license. Please check copyright information on all replacement figures and update the figure caption with source information. If applicable, please specify in the figure caption text when a figure is similar but not identical to the original image and is therefore for illustrative purposes only.

**RESPONSE**: We have requested the permissions from the relevant parties and attached the forms to our submission. The relevant copyright caption has also been applied.

6. We note that Figure 6 in your submission contain map images which may be copyrighted. All PLOS content is published under the Creative Commons Attribution License (CC BY 4.0), which means that the manuscript, images, and Supporting Information files will be freely available online, and any third party is permitted to access, download, copy, distribute, and use these materials in any way, even commercially, with proper attribution. For these reasons, we cannot publish previously copyrighted maps or satellite images created using proprietary data, such as Google software (Google Maps, Street View, and Earth). For more information, see our copyright guidelines: <http://journals.plos.org/plosone/s/licenses-and-copyright>.

We require you to either (1) present written permission from the copyright holder to publish these figures specifically under the CC BY 4.0 license, or (2) remove the figures from your submission:

A. You may seek permission from the original copyright holder of Figure 6 to publish the content specifically under the CC BY 4.0 license.

We recommend that you contact the original copyright holder with the Content Permission Form (<http://journals.plos.org/plosone/s/file?id=7c09/content-permission-form.pdf>) and the following text:

“I request permission for the open-access journal PLOS ONE to publish XXX under the Creative Commons Attribution License (CCAL) CC BY 4.0 (<http://creativecommons.org/licenses/by/4.0/>). Please be aware that this license allows unrestricted use and distribution, even commercially, by third parties. Please reply and provide explicit written permission to publish XXX under a CC BY license and complete the attached form.”

Please upload the completed Content Permission Form or other proof of granted permissions as an ""Other"" file with your submission.

In the figure caption of the copyrighted figure, please include the following text: “Reprinted from [ref] under a CC BY license, with permission from [name of publisher], original copyright [original copyright year].”

B. If you are unable to obtain permission from the original copyright holder to publish these figures under the CC BY 4.0 license or if the copyright holder’s requirements are incompatible with the CC BY 4.0 license, please either i) remove the figure or ii) supply a replacement figure that complies with the CC BY 4.0 license. Please check copyright information on all replacement figures and update the figure caption with source information. If applicable, please specify in the figure caption text when a figure is similar but not identical to the original image and is therefore for illustrative purposes only.

The following resources for replacing copyrighted map figures may be helpful:

USGS National Map Viewer (public domain): <http://viewer.nationalmap.gov/viewer/>

The Gateway to Astronaut Photography of Earth (public domain): <http://eol.jsc.nasa.gov/sseop/clickmap/>

Maps at the CIA (public domain): <https://www.cia.gov/library/publications/the-world-factbook/index.html> and <https://www.cia.gov/library/publications/cia-maps-publications/index.html>

NASA Earth Observatory (public domain): <http://earthobservatory.nasa.gov/>

Landsat: <http://landsat.visibleearth.nasa.gov/>

USGS EROS (Earth Resources Observatory and Science (EROS) Center) (public domain): [http://eros.usgs.gov/#](http://eros.usgs.gov/)

Natural Earth (public domain): <http://www.naturalearthdata.com/>

**RESPONSE**: We have replaced our GoogleMaps image with one of the open source maps suggested above.

7. Please upload a copy of Supporting Information Figure/Table/etc. “supplementary material 2” which you refer to in your text on page 7.

**RESPONSE**: Our supplementary material 2 has been renamed supplementary material 1 and added to our file.

[Note: HTML markup is below. Please do not edit.]

Reviewers' comments:

Reviewer's Responses to Questions

**Comments to the Author**

1. Is the manuscript technically sound, and do the data support the conclusions?

The manuscript must describe a technically sound piece of scientific research with data that supports the conclusions. Experiments must have been conducted rigorously, with appropriate controls, replication, and sample sizes. The conclusions must be drawn appropriately based on the data presented.

Reviewer #1: Yes

Reviewer #2: No

**RESPONSE**: The Reviewer incorrectly states this paper “outlines the methodology of a randomized controlled trial for delivering remote hearing and tinnitus therapy”. However, this is not correct as the paper outlines the learnings of delivering the first decentralised control trial for an ENT research topic. The results are presented showing new data, not only a methodology, and these results are discussed later in the paper as part of the wider literature.

2. Has the statistical analysis been performed appropriately and rigorously?

Reviewer #1: Yes

Reviewer #2: No

**RESPONSE**: The appropriate analysis of the data is presented in our results section.

3. Have the authors made all data underlying the findings in their manuscript fully available?

The [PLOS Data policy](http://track.editorialmanager.com/CL0/http:%2F%2Fwww.plosone.org%2Fstatic%2Fpolicies.action%23sharing/1/010f0195882786d5-85ee1977-8e8c-4401-aec2-f85b47854d50-000000/xnObxlGxJ39lrlQ-h0IHXQKe03xyQIgzr96Moz8JASY=202) requires authors to make all data underlying the findings described in their manuscript fully available without restriction, with rare exception (please refer to the Data Availability Statement in the manuscript PDF file). The data should be provided as part of the manuscript or its supporting information, or deposited to a public repository. For example, in addition to summary statistics, the data points behind means, medians and variance measures should be available. If there are restrictions on publicly sharing data—e.g. participant privacy or use of data from a third party—those must be specified.

Reviewer #1: Yes

Reviewer #2: Yes

**RESPONSE**: No comment

4. Is the manuscript presented in an intelligible fashion and written in standard English?

PLOS ONE does not copyedit accepted manuscripts, so the language in submitted articles must be clear, correct, and unambiguous. Any typographical or grammatical errors should be corrected at revision, so please note any specific errors here.

Reviewer #1: Yes

Reviewer #2: Yes

**RESPONSE**: No comment

5. Review Comments to the Author

Please use the space provided to explain your answers to the questions above. You may also include additional comments for the author, including concerns about dual publication, research ethics, or publication ethics. (Please upload your review as an attachment if it exceeds 20,000 characters)

Reviewer #1: Overall, a well structured manuscript with logical arguments for decentralized clinical trials, especially with hearing evaluation. Please clarify whether the trial use a stratified or unstratified randomization 1:1 ration. What statistical analytic methods were used to analyze the results. The manuscript will benefit with a more detailed discussion on the limitations of decentralized trials, including potential biases for research.

**RESPONSE**: We appreciate the comments made by Reviewer 1.

We can confirm stratified randomisation 1:1 ratio was implemented, and this has been integrated into the methodology in the main text.

Regarding the statistical analysis, our data is presented as percentages and proportions in our results section. Complex statistical analysis was not required as part of the data analysis regarding the study feasibility and role of decentralised randomised control trials. The learnings from the data is useful to the medical community.

We rebut the comment regarding “The manuscript will benefit with a more detailed discussion on the limitations of decentralized trials” as there is a sizeable section outlining the Limitations of decentralised studies which is labelled as such on line 369.

Reviewer #2: Dear authors,

This paper outlines the methodology of a randomized controlled trial for delivering remote hearing and tinnitus therapy. However, it closely resembles a previously published study protocol, "Digital Therapy for Improved Tinnitus Care Study (DEFINE): Protocol for a Randomised Controlled Trial."

The primary concern is that this paper does not present data on the treatment itself. Instead, it focuses on lessons learned from the study, which is more reflective of a report rather than a research paper.

**RESPONSE**: We appreciate the comments made by Reviewer 2 however disagree regarding the similarity of our paper with the protocol study quoted and that the paper is a reflective report.

The study protocol that has been previously published outlines what the study had planned to complete. This paper provides new information on study feasibility of a new methodology of decentralised randomised control trials in a field where it has not been previously used. It provides novel data that provides benefit to the medical community in addition to that of the published protocol.

We also disagree that the paper does not present data to warrant being a research paper. The paper outlines the lessons learnt from a novel methodology and are not a reflective account of the study. Data on the outcomes of the study methodology is provided and demonstrates the feasibility of the decentralised randomised control trials.
